# Paralysis in a Lion

**Published:** 1896-07

**Authors:** Cecil French

**Affiliations:** Washington, D. C.


### PARALYSIS IN A LION.

BY CECIL FRENCH, D.V.S.,
WASHINGTON, D. C.

THE proprietor of a travelling menagerie temporarily located in Washington recently sought my services for the relief of a male lion, aged about ten months. The condition was one of almost complete paralysis. The jaws and throat were the only organs over which he seemed to have control. The former were mostly opened, though he would occasionally close them, whilst the latter he moved continuously as if in the act of swallowing. The rectal temperature varied that day from 101.5° to 100.3°. Pulse ranged from 130 to 120. Smith's *Physiology of the Domestic Animals* gives a table compiled by Colin, in which the frequency of the pulse of the lion is placed at 40, that of the lioness at 68. In order to verify these citations I obtained both temperature and pulse-rate in a healthy male and female of the same litter, which were as follows: Male (ten months), pulse 110; temperature 100.° Female (ten months), pulse 120; temperature 102°. The observations were repeated with almost similar results and with due regard to absence of excitement. Making necessary allowance for difference in rate according to age (a lion is mature at seven years) there would seem to be considerable discrepancy between these tables.

I suspected the presence of intestinal parasites, and accordingly administered with some difficulty 10 grains of santonin. I was unable to make a visit the next day, but on that following was told by the proprietor and attendants that "millions of maggots had been passed, some living, but most of them dead, and that they resembled those infesting cheese." None had been preserved and a further dose did not bring any more away. Being a little dubious of this statement I procured from another lion some ascarides and asked if there was any resemblance. They were positive there was none, and then went on to describe them as being "whitish, with slightly colored head, short, thick body, and some had crawled about the host's body after excretion."

From other explanations and remembering that santonin had little if any effect on tapeworm, I excluded proglottides from consideration. I then asked to be shown the meat that was being fed. This I found considerably fly-blown, and they told me that it was often in that condition.

I therefore came somewhat unwillingly to the conclusion that the paralysis had been brought about reflexly by an obstruction of fly-maggots hatched after ingestion of fly-blown meat or by absorption of ptomaïnes secreted by the same. At any rate, from the day the animal was delivered of the pest he commenced to steadily improve, and a fortnight later began to make the administration of his medicine—extract of nux vomica in 1-grain doses, twice daily—a lively matter. I inquired of Dr. Howard, entomologist at the Bureau of Animal Industry, his opinion as to the veracity of the statements from the men and the possibility of the phenomenon. He was of opinion that hatching and development of the larvæ could take place under the conditions described.

